# Diamond Nanowires: A Novel Platform for Electrochemistry and Matrix-Free Mass Spectrometry

**DOI:** 10.3390/s150612573

**Published:** 2015-05-27

**Authors:** Sabine Szunerits, Yannick Coffinier, Rabah Boukherroub

**Affiliations:** Institute of Electronics, Microelectronics and Nanotechnology (IEMN), UMR-CNRS 8520, Université Lille 1, Avenue Poincaré—BP 60069, 59655 Villeneuve d’Ascq, France; E-Mails: yannick.coffinier@iri.univ-lille1.fr (Y.C.); rabah.boukherroub@univ-lille1.fr (R.B.)

**Keywords:** diamond nanowires, diamond nanostructures, synthetic methods, electrochemical sensing, mass spectrometry, SALDI

## Abstract

Over the last decades, carbon-based nanostructures have generated a huge interest from both fundamental and technological viewpoints owing to their physicochemical characteristics, markedly different from their corresponding bulk states. Among these nanostructured materials, carbon nanotubes (CNTs), and more recently graphene and its derivatives, hold a central position. The large amount of work devoted to these materials is driven not only by their unique mechanical and electrical properties, but also by the advances made in synthetic methods to produce these materials in large quantities with reasonably controllable morphologies. While much less studied than CNTs and graphene, diamond nanowires, the diamond analogue of CNTs, hold promise for several important applications. Diamond nanowires display several advantages such as chemical inertness, high mechanical strength, high thermal and electrical conductivity, together with proven biocompatibility and existence of various strategies to functionalize their surface. The unique physicochemical properties of diamond nanowires have generated wide interest for their use as fillers in nanocomposites, as light detectors and emitters, as substrates for nanoelectronic devices, as tips for scanning probe microscopy as well as for sensing applications. In the past few years, studies on boron-doped diamond nanowires (BDD NWs) focused on increasing their electrochemical active surface area to achieve higher sensitivity and selectivity compared to planar diamond interfaces. The first part of the present review article will cover the promising applications of BDD NWS for label-free sensing. Then, the potential use of diamond nanowires as inorganic substrates for matrix-free laser desorption/ionization mass spectrometry, a powerful label-free approach for quantification and identification of small compounds, will be discussed.

## 1. Introduction

**Figure 1:**
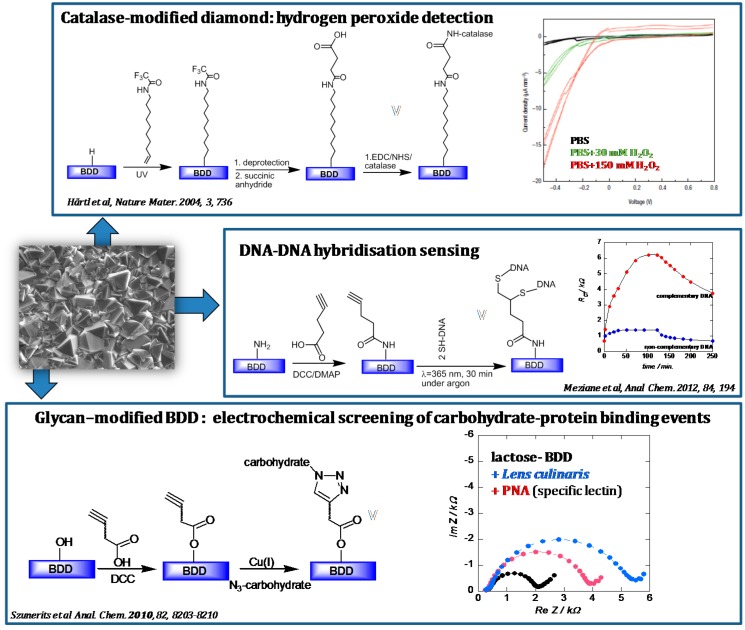
Selected examples of electrochemical sensing using BDD electrodes.

Diamond, a natural as well as a synthetic material, has captured researchers’ attention since decades. From any list summarizing the specific material properties, diamond is often at the extreme [1]: crystalline diamond shows the highest atomic density of any bulk crystal, the highest bulk modulus and the highest thermal conductivity. Diamond, a wide band gap semiconductor, is optically transparent from the far infrared to the ultraviolet, making it an ideal candidate for optical applications. During the growth of diamond films using chemical vapor deposition (CVD) systems, dopants and impurities can be readily incorporated into the material, allowing for tuning its optical and electrical properties. Substantial progress has been made in this area using boron for *p*-type doping and heavily boron-doped diamond (BDD) films (B-doping levels > 10^20^ cm^−3^) are now produced routinely for electrochemical investigations. The interest of BDD films for electrochemical sensing arises from the wide potential window and negligible capacitive current achieved as well as their stability required for use in *in vitro* biosensing applications [2,3,4,5,6,7]. Figure 1 summarizes some examples of BDD applications in electrochemical sensing.

**Figure 2:**
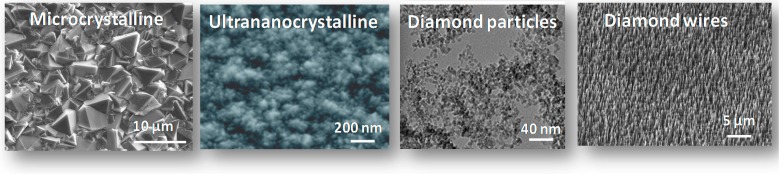
Different morphologies and forms of diamond.

The attractiveness of diamond is that different morphologies and forms can be obtained from this sp^3^ hybridized material (Figure 2). Indeed, modulation of the growth parameters results in microcrystalline to ultrananocrystalline CVD diamond films. Ultrananocrystalline films have the advantage of smooth surfaces, lower strain and improved fracture resistance. Such films are characterized by diamond domains that are ≈10 nm or less in size with thin sp^2^-bonded boundaries. 

Nanoscale diamond particles (also termed nanodiamonds, NDs) represent another interesting form widely explored for applications in drug delivery or medical diagnostics. The first records of the production of NDs date back to the 1960s, when a group of Soviet scientists discovered single crystals of cubic diamond particles in soot produced by detonating an oxygen-deficient TNT/hexogen composition in inert media without using any extra carbon source [8]. Since then, NDs have been rapidly gaining popularity in bioimaging and drug delivery applications, since colloidal suspensions of individual diamond particles with diameter of 4–10 nm are commercially available.

Diamond in the form of nanowires has to be added to this list. The use of diamond nanowires is believed to address positively issues related to improving the overall performance of sensors, including sensitivity and selectivity [9,10,11,12]. For a long time, the routine use of diamond nanowires was restricted, as no viable methods for their fabrication were available. The first attempt to synthesize diamond nanowires dates back to 1968 using a radiation heating unit developed from a super-high pressure Xenon lamp [13]. Only limited progress in this direction was made due to the difficulty of controlling the dimension of the diamond filament and lack of characterization facilities at that time, restricting further investigation of such diamond-based whiskers. 

The intent of this review article is to make the reader more familiar with recent developments for the preparation of diamond nanostructures and their use for label-free sensing applications. The different synthetic methods can be classified into two main approaches: “top-down” and “bottom-up” approaches. Due to their high surface area, boron-doped diamond nanowires (BDD NWs) represent an interesting platform for electrochemical sensing as compared to planar BDD electrodes. This will be demonstrated in various examples of electrochemical sensing of different chemical/biological species using BDD NWs. Finally, the potential applications of diamond nanowires as inorganic matrix for surface-assisted laser desorption/ionization mass spectrometry (SALDI-MS) will be discussed in details. 

## 2. Synthetic Routes of Diamond Nanowires

Reports on the fabrication of diamond structured surfaces with diameters as small as 25 µm and hundreds of microns in length date back to the 1960s [13]. However, it was only around the beginning of the 21st century that further attempts for the synthesis of diamond nanostructures were undertaken. The main approaches for the successful fabrication of diamond nanostructures are generally based on “top-down” and “bottom-up” processes. 

### 2.1. Top-Down Approach

**Figure 3:**
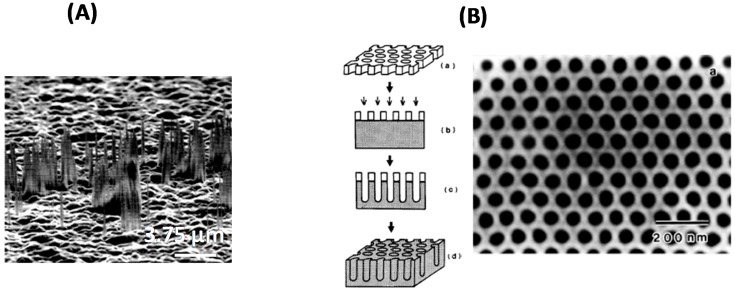
(**A**) SEM images of diamond nanowiskers formed on as-grown diamond films coated with 400 nm thick Al layer using reactive ion etching (RIE) with oxygen plasma (reprint with permission from [16]); (**B**) Formation of honeycomb diamond films (reprint with permission from [15]).

One of the initial attempts for a top-down synthesis of diamond nanostructures was reported by Shiomi [14]. Reactive ion etching (RIE) with oxygen plasma of CVD diamond film coated with a 400 nm thick aluminum layer resulted in columnar structures of approximately 300 nm in length and 10 nm in diameter (Figure 3A) [14]. The plasma-assisted RIE technology has since then been widely investigated for the top-down fabrication of diamond nanowires and pillars. Masuda *et al.* proposed, for example, the use of porous anodic aluminum oxide masks for the formation of diamond honeycomb films via oxygen plasma etching of CVD diamond films through the holes of porous alumina films (Figure 3B) [15]. They showed that the etching rate of alumina, compared to that of the diamond film, is negligible, resulting in the formation of honeycomb structures with high aspect ratios. Uniform holes with an average diameter of 70 nm and spacing of 100 nm with uniform depth of ~0.6 µm have been etched perpendicular to the diamond film surface, yielding an aspect ratio of ~9. An important fact is that this lithographic process was carried out in non-contact mode: the mask is merely placed on the substrate and therefore does not adhere to the substrate surface, unlike the situation with the resist-type masks commonly used in lithography. This makes the approach fast and easy to perform on different diamond interfaces.

**Figure 4:**
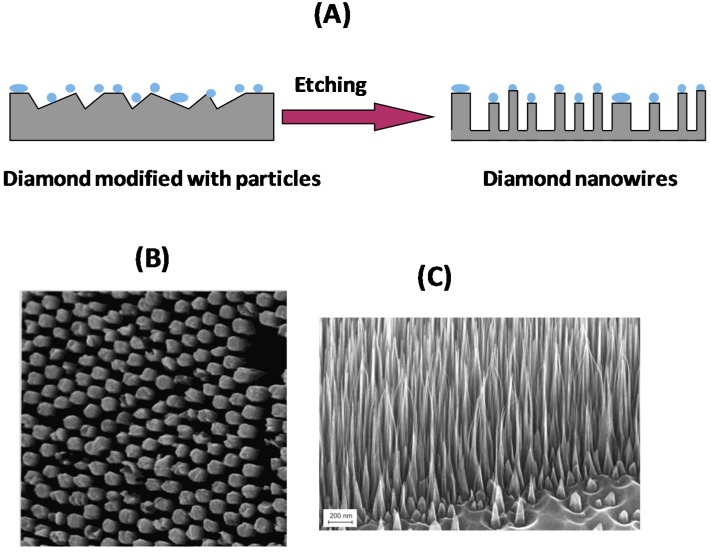
(**A**) Top-down etching process of CVD diamond using seed particles; (**B**) SEM image of diamond cylinders obtained using oxygen reactive ion etching (RIE) for 60 min through a 1 µM SiO_2_ particle array (reprint with permission from [18]); (**C**) SEM image of vertically aligned diamond nanowires using diamond nanoparticles as masks (with courtesy of C. Nebel).

Beside such masks, arrays of nanoparticles, seeded onto CVD grown diamond films, have been investigated by several groups as attractive alternatives. Aluminum [17], SiO_2_ [18], gold [19], as well as diamond nanoparticles [11,20] proved to be useful etching masks (Figure 4A). Okuyama *et al.* used RIE with oxygen plasma through a two-dimensionally ordered SiO_2_ particle array to form diamond cylinders [18]. The diameter and the length of the cylinders depend on the etching time and vary between 0.6–1 µm in diameter and 3–4 µm in length (Figure 4B) [18]. High-density and uniform diamond nanopillar arrays were obtained by Zou *et al.* by employing bias-assisted RIE in a hydrogen/argon plasma using gold nanoislands of 150 nm in diameter as etching masks [19]. The gold islands protect the underlying diamond from etching and sputtering; nanopillars with gold clusters at the tip are produced. Gold nanoparticles were indeed found to be one of the most suitable seeding masks as they are easy to disperse, resulting in single nanoparticles on the surface requiring no further processing step. The etch rate of the gold nanoparticles mask is 25 nm/min [21]. This yields good etch selectivity and diamond wires of 900 nm in height, and diameters from 275 nm at bottom to 310 nm at the top can be produced [21].

The use of nanodiamond (ND) particles as a hard mask for RIE of diamond was examined by research groups at the AIST (Japan) and IAF (Germany) [11,20,22,23]. Vertically aligned diamond nanowires were obtained using RIE in an O_2_/CF_4_ (97/3%) gas mixture for etching times of 10 s (Figure 4C). The length of the wires was limited by simultaneous etching of the ND particles mask with an etching rate of 10 Å·s^−1^.

**Figure 5:**
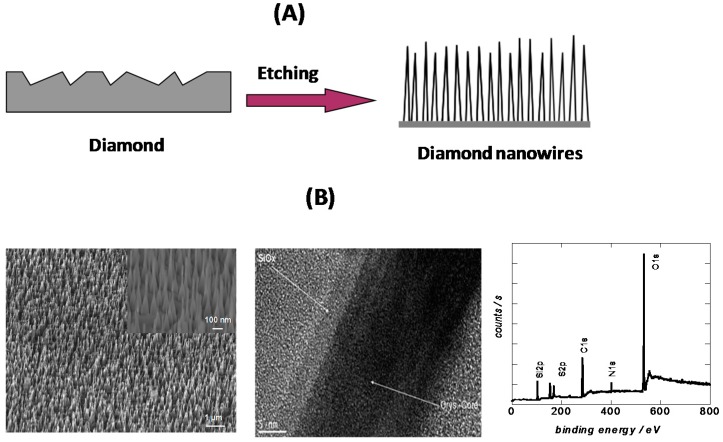
(**A**) Top-down etching process of CVD diamond without mask; (**B**) SEM and HR-TEM images, and XPS survey spectrum of boron-doped diamond nanowires synthesized through maskless technique (reprint with permission from [26]).

Mask-less top-down approaches have been recently proposed as alternatives [16,24,25,26,27] (Figure 5A). Such methods have the intrinsic advantage of being simple and straightforward, not requiring complicated processing steps such as mask deposition or template removal. Our group demonstrated that diamond nanowires can be easily prepared from highly boron-doped microcrystalline diamond thin films by RIE in an oxygen plasma (Figure 5A) [12,25,26,27]. The resulting nanowires are 1.4 ± 0.1 µm long with a tip and base radius of *r*_tip_ = 10 ± 5 nm and *r*_base_ = 40 ± 5 nm, respectively. The nanowires are about 140 times longer than aligned diamond nanowires prepared using diamond nanoparticles as a hard mask. X-ray photoelectron spectroscopy (XPS) analysis of the chemical composition of the diamond nanowires revealed that next to C_1s_ at 285 eV and O_1s_ at 532 eV, additional peaks at 402, 104 and 169 eV due respectively to N_1s_, Si_2p_ and S_2p_ are present in the spectrum (Figure 5B). The latter elements are believed to originate from surface contamination during the RIE process. Indeed, the presence of SiO_x_ shell around the BDD NWs was confirmed by HR-TEM analysis (Figure 5B). SiO_x_ was most likely deposited during the etching process due to sputtering of the substrate holder or the silicon wafer onto which the diamond film was deposited. A similar behavior was observed by Baik *et al.* when Mo sample holder was used [16]. The SiO_x_ deposits can be easily removed through immersion in HF aqueous solutions. 

### 2.2. Bottom-Up Approach

**Figure 6:**
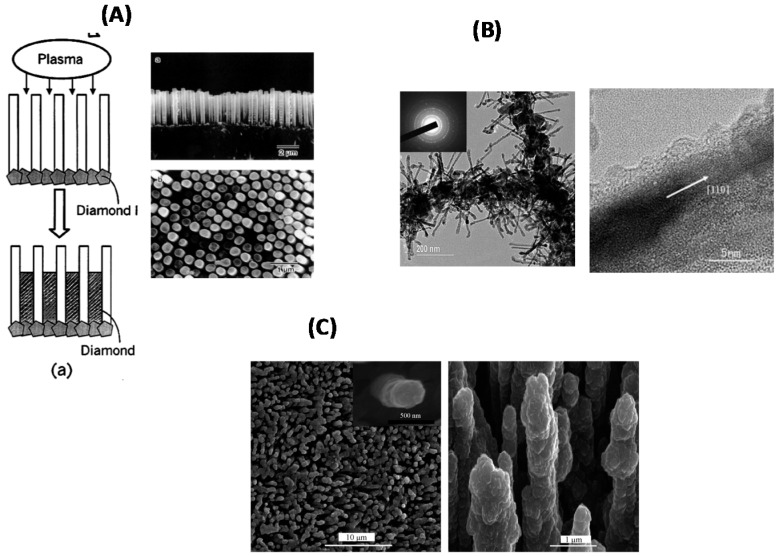
(**A**) Schematic diagram of the bottom-up fabrication of cylindrical diamond wires in porous alumina template (**left**) and the corresponding SEM images (reprint with permission from [28]); (**B**) TEM (**left**) and HR-TEM (**right**) images of diamond rods grown on carbon nanotubes (reprint with permission from [29]); (**C**) SEM image of diamond coated silicon nanowires (reprint with permission from [10]).

As the formation of diamond nanowires by the top-down approach is often accompanied with surface damage, whose impact is generally greater at the nanoscale due to the large surface-to-volume ratio, bottom-up procedures were also investigated in the literature. One of the first bottom-up approaches was described by Masuda *et al.* (Figure 6A) [28]. In this technique, microcrystalline diamond nanocylinders were grown on anodic oxide templates using microwave plasma-assisted CVD and 50 nm nanodiamond particles as seeds. The density of the obtained wires was as high as 4.6 × 10^8^ cylinders/cm^2^ with a wire length of about 5 µm and 300 nm in diameter (Figure 6A). Diamond nanorods of 8–10 nm in diameter and up to 200 nm in length coated with an amorphous carbon layer were grown along the (110) direction upon applying a prolonged hydrogen plasma to multi-walled carbon nanotubes (MWCNTs) (Figure 6B) [29]. The authors suggested that initial diamond nuclei can be formed at defect sites of the MWCNTs due to the presence of hydrogen. At high temperature (1000 K) in the presence of hydrogen, MWCNTs transform to amorphous material, where the nucleation of diamond phase is facilitated. The addition of N_2_ into the growth mixture of ultrananocrystalline diamond was reported by Vlasov and co-workers to change the surface morphology to wire-like structures [30]. The addition of 25% of N_2_ to the Ar/CH_4_/H_2_ gas mixture resulted in the formation of hybrid diamond-graphite nanowires with lengths up to a few hundred nanometers. This hybrid material consists of a single crystalline diamond core of 5–6 nm in diameter oriented along the (110) principal axis and graphitic shells of different thicknesses covering the core. Using a mixture of N_2_ and CH_4_ allowed also the growth of ultrathin diamond nanorods by microwave-assisted CVD [31]. The resulting nanorods exhibited a diameter as small as 2.1 nm, which is not only smaller than any other reported diamond nanostructures but also smaller than the theoretical value of energetically stable diamond nanorods. More recently, the synthesis of straight, thin and long diamond nanowires using atmospheric-pressure chemical vapor deposition was proposed [32,33]. The diamond nanowires showed a uniform diameter of 60–90 nm with over tens of micrometers in length. Spectroscopic analysis provided information that these nanowires are diamond with high crystallinity and high structural uniformity. 

Post-coating of preformed silicon nanowires by diamond thin films has attracted considerable interest in the past five years for the preparation of core-shell nanowires [10,34,35]. Boron-doped diamond nanoforest electrode could be fabricated on pre-formed silicon nanowires as illustrated in Figure 6C [10]. Silicon nanowires (Si NWs) were formed according to a procedure reported by Peng *et al.* [36], and subsequently post-coated with boron-doped diamond thin films deposited by high frequency CVD technology. The coverage of the nanocrystalline diamond film is complete and continuous along the whole length (5 µm) of the Si NWs as seen in the SEM images in Figure 6C. A similar strategy was employed by Gao and co-workers to coat Si NWs with a 100 nm layer of nanocrystalline diamond by microwave-enhanced CVD [34]. The grafting of negatively-charged diamond nanoparticles over cationic polymer-coated nanometric patterns was proposed by Girard and co-workers as a bottom-up strategy towards 3D diamond nanostructures [35].

## 3. Applications of Diamond Nanowires

**Figure 7:**
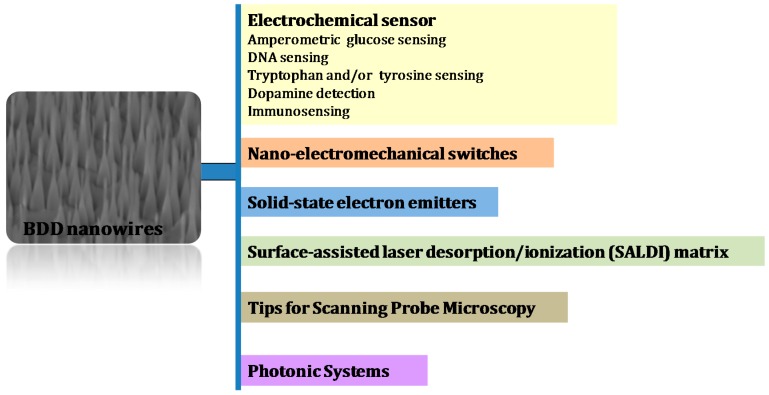
Applications of diamond nanowires.

In general, the synthesis of diamond nanostructures has been advanced to a high level in a very short time span. The access to such nanostructures allowed finally the use of diamond nanowires for different applications ranging from solid-state electron emitters, high performance nano-electromechanical switches, probes for scanning probe microscopy and photonic systems, to the formation of superhydrophobic and oleophobic interfaces [15,21,26,31,33,37] (Figure 7). The use of boron-doped diamond nanowires has in particular found interest for electrochemical sensing [10,11,12,25,38,39,40,41,42,43] and as surface-assisted laser desorption/ionization (SALDI) matrix [27]. 

### 3.1. Diamond Nanowires for Electrochemical Sensing

Diamond nanowires are among the fairly new but promising materials for chemical and biochemical sensing. The common theme of diamond sensors is that they convert biological or chemical information into an electrical signal, which can be measured accurately using a panel of electrochemical methods (e.g., cyclic voltammetry, differential pulse voltammetry, electrochemical impedance spectroscopy, *etc.*). As the technology required to create electrochemical biosensors is much cheaper than that required for fluorescence-based sensors, electrochemical sensors are dominating the analytical field. Their label-free character adds to their general value. While the conversion of a biological interaction to an electrical signal is attractive for sensors that are in continuous use or need to withstand harsh environments, so far, electrochemical sensors are in general several orders of magnitude less sensitive than the best fluorescence-based detection sensors. 

**Figure 8:**
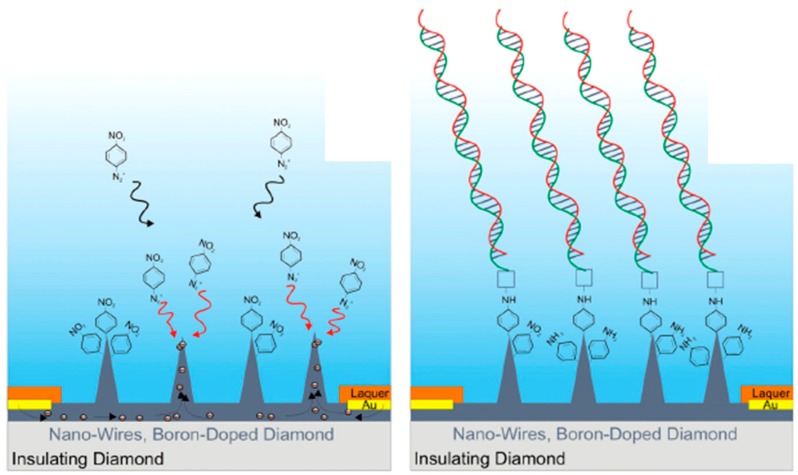
Diamond nanowires for DNA sensing: Preferential linking of phenyl aryl and DNA molecules to the tip of the wires (reprint with permission from [23,44]).

Yang *et al.* were the first to demonstrate that the detection limit of electrochemical biosensors can be markedly improved if vertically aligned diamond nanowires are used [11]. DNA sensors were prepared through immobilization of single strand DNA probes onto diamond nanowires pre-functionalized with amine-terminated phenyl groups in an electrochemical functionalization step (Figure 8) [23,44]. The enhanced electrical field at the very end of the diamond tips resulted in a preferential DNA alignment at the tip rather than at the walls of the wires, increasing the probes’ accessibility for interaction. This gave rise to optimized hybridization kinetics of complementary DNA (cDNA) and high sensitivity with a detection limit of 2 pM for cDNA as well as single-base mismatch discrimination. 

**Figure 9:**
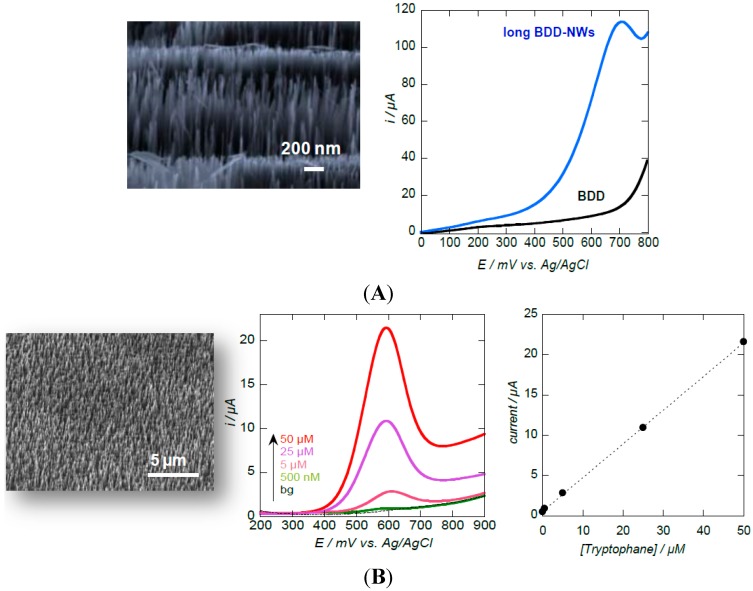
Diamond nanowires for enzyme-free glucose and tryptophan sensing: (**A**) SEM image of long diamond nanowires produced through mask-less RIE of CVD diamond films and linear sweep voltammogram recorded in 2 mM glucose solution (0.1 M NaOH) for BDD (black) and long BDD NWs (blue) (reprint with permission from [12]); (**B**) SEM image of short diamond nanowires together with differential pulse voltammograms for different concentrations of tryptophan and the corresponding calibration curve (reprint with permission from [25]).

Diamond nanowires electrodes allow also the direct electrochemical detection of glucose under strong basic conditions [10,12,45]. While almost no visible anodic peak for glucose oxidation was observed during the positive potential scan on a planar BDD electrode, a well-defined current response for glucose was obtained, for example, on BDD NWs electrodes of ≈3 µm in length with a diameter ranging from 10–50 nm [12]. The detection limit of this sensor was 60 µM (Figure 9). Such an improvement in glucose oxidation suggests that the Faradaic current of glucose oxidation depends strongly on the surface structure and porosity of the electrode, and the accessible surface area. The results reported by Nebel and co-workers showed that decoration of diamond nanopillar electrodes with Ni-nanoparticles improves the sensitivity of the sensor for glucose detection with a detection limit of 10 µM [45]. Diamond coated Si nanowires were investigated by Luo *et al.* for glucose sensing with an estimated detection limit of 0.2 µM and a sensitivity of 8.1 µA·mM^−1^·cm^−2^ [10].

Diamond nanowires are also adapted for the sensitive electrochemical detection of aromatic amino acids such as tryptophan and tyrosine, two important precursors of adrenaline, dopamine or melatonine [12,25]. A detection limit of 5 × 10^−7^ M was recorded for tryptophan on BDD NWs electrodes [25], being significantly lower than on planar microcrystalline BDD (1 × 10^−5^ M) [46]. The simultaneous detection of tryptophan and tyrosine by differential pulse voltammetry is also possible on BDD NWs electrodes, when the amount of tryptophan present in the mixture is not exceeding tryptophan/tyrosine ≤ 0.5 [41]. The oxidation of other small molecules such as dopamine, uric acid and ascorbic acid was reported by Shalini *et al.* using nitrogen-doped diamond nanowire electrodes [47].

**Figure 10:**
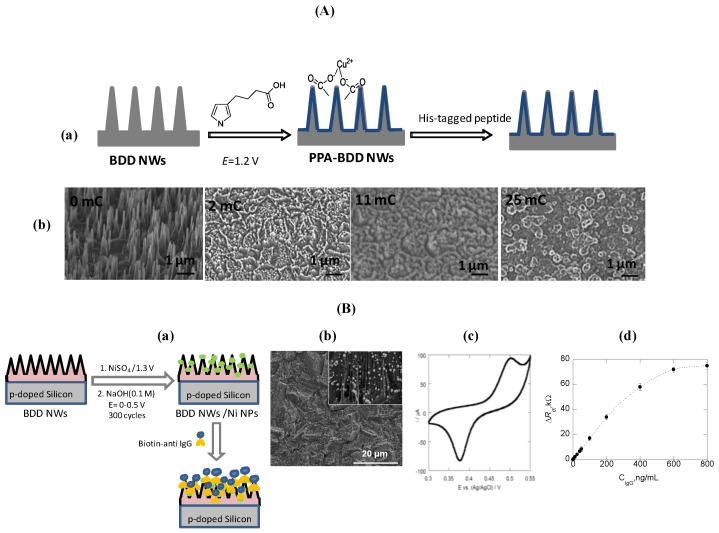
Diamond nanowires for immunosensing. (**A**) (**a**) fabrication method of polymer coated BDD NWs (PPA BDD NWs) with chelated Cu^2+^ ions and subsequently modified with histidine-terminated target; (**b**) SEM images of BDD NWs after electrochemical deposition of carboxylic acid-terminated poly(pyrrole) (100 mM) in TBATFB (0.1 M)/ acetonitrile solution at *E* = +1.2 V for different deposition charges (2, 11, 23 mC·cm^−2^) (reprint with permission from [39]); (**B**) Fabrication method of Ni NPs modified diamond nanowires (**a**), SEM image of Ni NPs modified diamond nanowires (**b**), CV of Ni NPs-BDD NWs in 0.1 M NaOH (**c**), Calibration curve for IgG (**d**) (reprint with permission from [40]).

Electrochemical immunosensors based on chemically modified BDD NWs electrodes have been lately developed by our group [38,39,40]. Diamond nanowires immunosensors were constructed by coating diamond nanowires with functional conducting polymer films (e.g., carboxylic acid-terminated poly(pyrrole), copper ion (Cu^2+^) chelation followed by linkage of histidine-tagged peptides [39]) (Figure 10A) or by electrochemical deposition of nickel nanoparticles (Ni-NPs) onto diamond nanowires followed by immobilization of biotin-tagged anti-IgG (Figure 10B**)** [38,40]. Post-coating of diamond nanowires with polymer films can be achieved by amperometrically biasing diamond nanowire electrodes at 1.2 V *vs.* Ag/AgCl in 3-(pyrrole) carboxylic acid solution [39]. Fine-tuning the charge allowed coating the wires rather than the formation of polymer films (PPA) in solution. Figure 10A shows SEM images of BDD NWs coated with carboxylic acid-terminated polypyrrole (PPA-BDD NWs) by varying the deposition time. At very low deposition charge (2 mC·cm^−2^), the polymer started to form preferentially at the defect sites of the interface *i.e.*, in-between the BDD NWs. Increasing the deposition charge to 11 mC·cm^−2^ resulted in polymer coated BDD NWs, while large deposition charges (23 mC·cm^−2^ and higher) led to a loss of the wire structure in favor of continuous film formation. The available carboxylic groups of the poly(pyrrole) coated wire electrode allows their coordination with copper ion (Cu^2+^), known to be specific binding sites for His-tagged analytes. Indeed, the affinity constant (*K*_A_) of His-Tag-des-Arg^6^-Bradykinine peptide to Cu^2+^ coordinated carboxyl-terminated diamond wires was determined as *K*_A_ = (1.15 ± 0.5) × 10^6^ M^−1^, higher than that determined in the absence of Cu^2+^ (*K*_A_ = (0.31 ± 0.5) × 10^6^ M^−1^). Concentrations as low as 10 nM resulted in *R*_CT_ shift of 50 ± 22 Ω on these interfaces, while on a planar BDD interface modified with carboxylic groups and chelated with Cu^2+^, His-tagged peptide concentrations had to exceed 100 nM to cause a comparable shift. 

The other approach consists of the deposition of nickel nanoparticles. Figure 10B(b) shows a representative SEM image of Ni NPs modified diamond nanowires (BDD NWs/Ni NPs) revealing particles of 20 ± 5 nm in diameter and a particle density of 150 Ni NPs/µm^2^. The oxidation state of the deposited nickel was confirmed by the presence of a quasi-reversible redox peak at ≈0.47 V/Ag/AgCl in the cyclic voltammogram of the BDD NWs/Ni NPs electrodes in PBS (Figure 10B(c) assigned to Ni(OH)_2_/NiOOH. The hydrated nickel hydroxide can be present in two crystalline forms: the hydrated α-Ni(OH)_2_ and the anhydrous β-Ni(OH)_2_, the latter being the more stable and preferentially formed by cycling in NaOH (0.1 M) [48]. Biotinylated anti-IgG was specifically linked to the Ni^2+^ particles and the change in the interfacial properties upon binding of different concentrations of IgG was detected using electrochemical impedance spectroscopy. A linear relation between Δ*R*_ct_ and IgG concentration in the range of 0.3–400 ng/mL was recorded (Figure 10A(d)) with a correlation coefficient of r = 0.9996 according to Δ*R*_et_ (kΩ) = 0.02 + 0.0451 × [IgG]. The detection limit of IgG was determined to be ≈0.3 ng/mL from five blank noise signals (95% confidence level). The advantage of this approach is the possibility of a controlled immobilization of biotinylated antibodies and antigens with the omission of avidin layers, influencing the electrochemical behavior of the electrical interface. As the affinity of the biotin-tag to the Ni NPs is weaker than avidin-biotin interaction, an easy regeneration of the interface through a simple ethylenediaminetetraacetic acid (EDTA) wash was achieved. This could allow in the future the immobilization of different antibodies using the same interface.

### 3.2. Diamond Nanowires for Matrix-Free Mass Spectrometry

Mass spectrometry (MS) is widely accepted as a ‘gold-standard’ method for the identification of chemicals or biological products. It is, nowadays, applied in highly diversified domains like those directly or indirectly tied to healthcare or regulation-driven demand such as drug development, diagnostics, food and environmental safety testing. Among the different methodologies, matrix-assisted laser desorption/ionization mass spectrometry (MALDI-MS), first introduced in 1988 by Hillenkampf and Karas [49], constitutes one of the soft ionization techniques that provides the nondestructive vaporization and ionization of analytes using UV-absorbing organic matrices. The utility of MALDI-MS for protein and peptide analysis lies in its ability to provide high accurate molecular-weight information on intact molecules. Acquiring optimum MALDI data depends, however, not only on the functional and structural properties of the analyte itself but also largely on the choice of suitable matrices. As the matrix is the medium by which the analyte is transported to the gaseous phase and provides the conditions that makes ionization possible, the matrix and the sample-matrix preparation procedure greatly influence the quality of MALDI mass spectra. Organic matrices such as 2,5-dihydroxylbenzoic acid (DHB), α-cyano-4-hydroxycinnamic acid (HCCA) or sinapic acid (SA) are commonly used. However, the choice of the matrix remains an empirical issue. While MALDI-MS has been successfully used to analyze large molecules [50,51,52], it has rarely been applied to low-molecular weight compounds (<500 Da) as a large number of matrix ions appear in the low-mass range. 

**Figure 11:**
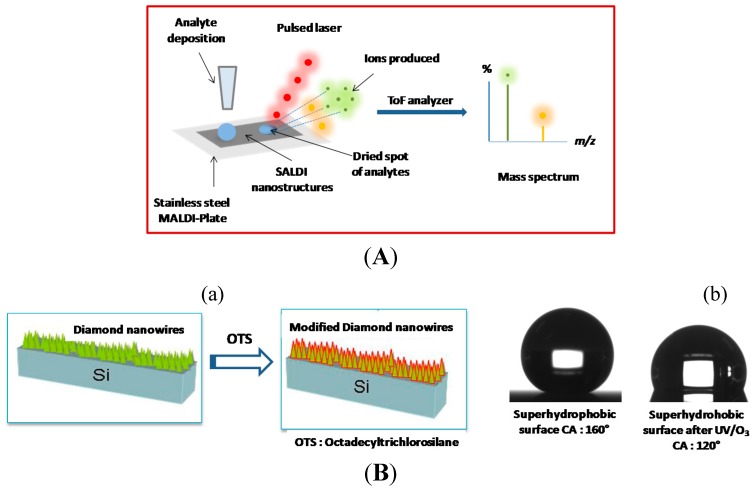

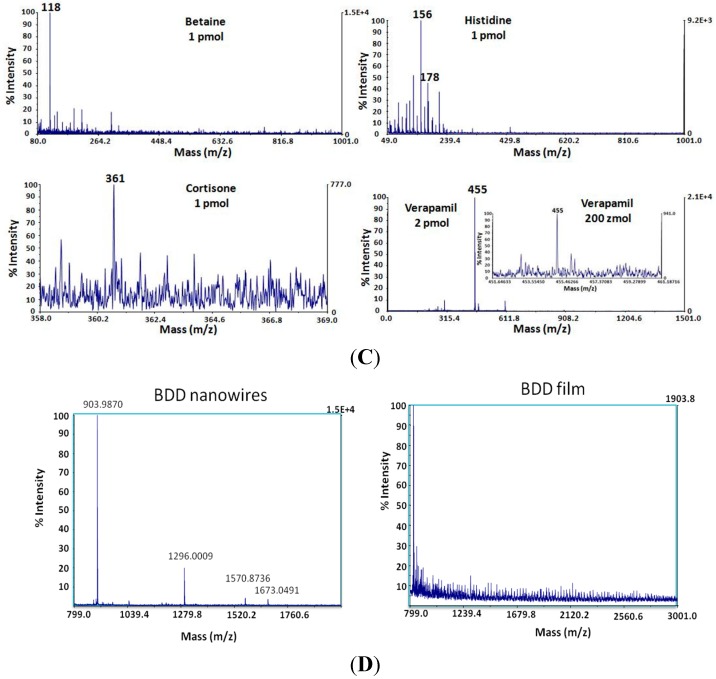
Diamond nanowires as inorganic matrices for SALDI. (**A**) Schematic presentation of the principle of surface-assisted laser desorption/ionization mass spectrometry (SALDI-MS) with analyte deposition on SALDI based nanostructures such as diamond nanowires; (**B**) OTS modified diamond nanowires (**a**) and contact angle values before and after UV/O_3_ treatment (**b**); (**C**) LDI-MS spectra on BDD NWs of various compounds (Histidine, *m*/*z* 156, 1 pmol; Betaine m/z 118, 1 pmol; Cortisone m/z 361, 1 pmol and verapamil m/z 455, 2 pmol and 200 zmol (inset)); (**D**) LDI-MS detection of a peptide mixture ([Des-Arg^1^]-bradykinin m/z 904 (50 fmol/µL), angiotensin I *m*/*z* 1296 (50 fmol/µL), [Glu^1^]-fibrinopeptide B m/z 1570 (50 fmol/µL), neurotensin *m*/*z* 1673 (10 fmol/µL)) on BDD NWs and BDD films (reprint with permission from [27]).

Tanaka and co-workers proposed the use of a suspension of cobalt nanoparticles of 30 nm in size mixed with glycerol as inorganic matrix for the laser desorption/ionization of analytes [53]. Since then, several alternative inorganic matrices have been proposed as assisting material [54] in a process that was named surface-assisted laser desorption/ionization mass spectrometry (SALDI-MS) by Sunner *et al.* [55]. The basic principle of SALDI-MS analysis is schematically outlined in Figure 11A. Using inorganic species as the assisting material in MALDI is an alternative approach to avoid the problems arising in conventional MALDI analysis using organic compounds as matrix systems [56]. Nanostructured diamond-like carbon (DLC) coated targets were proposed by Bonn and co-workers as versatile platform for the analysis of amino acids, carbohydrates, lipids, peptides and other metabolites using laser desorption/ionization mass spectrometry [57]. A nanodiamond MALDI-MS support was demonstrated by Wei *et al.* to improve the ionization efficiency of samples [58]. The use of boron-doped diamond nanowires as an inorganic matrix for the D/I of peptides and small molecules, and their analysis by mass spectrometry with a very high sensitivity has been demonstrated by us [27]. To minimize droplet spreading on the matrix surface, the nanowires were chemically functionalized with octadecyltrichlorosilane (OTS) to reach a final water contact angle of 120 °C (Figure 11B). The sub-bandgap absorption under UV laser irradiation and the heat confinement inside the nanowires allowed LDI-MS of various compounds, most likely via a thermal mechanism (Figure 11B). One of the most used compounds to assess the LDI-MS performances of SALDI surfaces is verapamil, a calcium channel blocker. Detection of verapamil on silicon nanowires, prepared by the Metal-Assisted Chemical Etching (MACE) method, has shown a detection limit value of 5 fmol [59]. Walker *et al.* explored silicon nanopost arrays (NAPA) in combination with LDI-MS. The high ionization efficiencies enabled detection of ultratrace amounts of analytes (800 zmol of verapamil) within a dynamic range spanning up to four orders of magnitude [60]. For comparison, the detection limit value of verapamil on diamond nanowires was 200 zeptomoles, which is slightly better. Impressive results were obtained by Northen *et al.* by nanostructure-initiator mass spectrometry (NIMS). They demonstrated a detection limit value for verapamil of 700 ymol [61]. Although this technique is not a “matrix” *sensu stricto* (as MALDI), NIMS takes advantage of an “initiator” compound (e.g., bis(tridecafluoro-1,1,2,2-tetrahydrooctyl)tetramethyldisiloxane), which assists desorption and/or ionization of analyte molecules. Even though the limit of detection (LoD) that is needed depends on the targeted application, most of the time, the sensitivity race is not scientifically or even technically relevant. Other parameters should be taken into account for performant SALDI surfaces such as laser fluence, dynamic range, versatility, peptide discrimination and post-translational modification (PTMs) detection (proteomics field), cost, reproducibility.

The investigated diamond nanowires, undoped (UDD NWs) or boron-doped (BDD NWs), display antireflective properties permitting photons absorption at λ = 337 nm. However, on the mass spectrum of a peptide mixture obtained on BDD NWs interface, all peptides have been detected with relatively high signal intensity (Figure 11D). On the contrary, the same experiment performed on a planar nanocrystalline BDD, *i.e.*, the same interface without any RIE step process etching, no peaks were observed. Thus, the absence of peaks in the MS spectrum (Figure 11D) clearly indicates that the presence of nanostructures on the BDD substrate is mandatory for achieving the laser desorption/ionization (LDI) of biomolecules [27].

### 3.3. Future Trend: Coupled Electrochemistry-Mass Spectrometry Analysis on Diamond Nanowires

Due to the great performance of BDD nanowires for electrochemical sensing and SALDI-MS, their combined use might provide a powerful quantitative and qualitative analysis platform. Electrochemistry coupled with mass spectrometry (EC/MS) allowed the identification of products or/and intermediates of electrochemical reactions, which is not only useful for elucidation of redox reaction mechanisms but also leads to many valuable bioanalytical applications [62,63]. The versatility of EC/MS stems from two facts. MS can serve as a sensitive and general detector for electrochemical compounds and can provide molecular weight information about an analyte of interest. In addition, tandem MS analysis can be used for structural determination based on ion dissociation. Electrochemical conversion, on the other hand, can improve analyte ionization and provides desired modification to the analyte prior to MS analysis. Attracted by the complementary nature of these two techniques, the coupling of EC and MS appears perfect and appealing. For more than four decades, researchers have been engaged in coupling these two techniques [64,65,66,67].

A key challenge of the coupling is how to interface an electrochemical cell online with a mass spectrometer for efficiently ionizing the electrolyzed samples. Therefore, selecting an appropriate ionization method is critical. In this regard, the evolution of the coupled EC/MS technique followed the advances of ionization methods in the field of mass spectrometry. In 1971, the first EC/MS device was introduced by Bruckenstein and Gadde for *in situ* mass spectrometric determination of volatile electrode reaction products [64]. In the experiment, a Teflon membrane was placed between the porous electrode and the mass spectrometer ionization chamber so that volatile reaction products could penetrate through the membrane and be ionized by electron impact (EI) without the interference of the solvent. In 1984, differential electrochemical mass spectrometry (DEMS) was established for the online MS detection of volatile electrochemical products in real time. The total response time was less than 1 s [68]. Furthermore in DEMS, the product MS signal intensity was proportional to the Faradaic current as only the volatile compounds produced were transferred to the ionization chamber, from which quantitation could be achieved. Then, several methods of ionization have been used including thermospray (TS), fast atom bombardment (FAB), inductively coupled plasma (ICP), chemical ionization (CI), atmospheric pressure chemical ionization (APCI), atmospheric pressure photoionization (APPI) and electrospray ionization (ESI). This former combination (EC/ESI-MS) was the most widely used for many applications [62]. Desorption electrospray ionization (DESI) or nanoDESI and direct analysis in real time (DART) are other recent methods to perform ambient soft ionization with little or no sample preparation [69]. Applications of EC/MS are various and numerous and among them we can cite mechanistic elucidation of electrochemical reactions [70,71,72,73], mimicking of metabolic pathways [65], tagging of protein/peptide thiol groups using various electrochemical generated species to electrochemically enhance MS signals [74,75], pre-concentrate target via electrochemical deposition [66], and following protein/peptide cleavage and online MS analysis that is very notably useful in the proteomics field [76,77].

## 4. Conclusions

From the above discussion, it becomes clear that a large amount of effort has been devoted to the synthesis of diamond nanostructures to a point where they can be considered for device-oriented applications. The discoveries and research undertaken in the last years hope to trigger the development of diamond nanowire sensors for clinical diagnostic, environmental sensing and other applications at the interface between biology, physics and chemistry. However, the full spectrum of such nanostructures for other technological applications cannot be overseen. Diamond coated silicon nanowires have been lately investigated for supercapacitor applications [34], bringing diamond nanostructures to the field of energy. A full and detailed understanding of the electrical and electrochemical properties of a single diamond nanowire might be of ultimate importance in the near future to foster further such developments.

The use of diamond nanowires is believed to have great potential for EC/MS. Indeed, coupling EC/MS using diamond nanowires will permit performing both electrochemistry and MS detection of ionized compounds achieved by either DESI (or nanoDESI) or the LDI ionization process. 

Surface functionalization is required for almost any kind of sensing applications. Currently, the reported surface functionalization schemes of diamond wires are limited to some examples. Widening this area is thus one aspect that should be undertaken by research groups working in this field. The formation of superhydrophobic and oleophobic interfaces has, for example, been demonstrated to have impact on cell and bacteria adhesion [26]. An important aspect will be the determination of the influence of diamond doping levels and even wire length on SALDI results. A better understanding will help to optimize the technique and achieve highly reproducible and accurate results. This will make the approach of high interest for any laboratory.
